# Watt’s Elevator—A Misnomer

**Published:** 1871-04

**Authors:** 


### "WATT'S ELEVATOR"—A MISNOMER.

In the advertising department of the *Dental Cosmos* for January, 1870, reference is made to an instrument named as above. Being totally ignorant of the existence of any such instrument, we ordered a few, on suspicion, as it was embarrassing not to be able to describe our own elevator. And now we propose a brief history of the name, and of the instrument.

After we had retired from practice, we showed a favorite instrument to our friend, Dr. B. F. Arrington, and if by any intimation we impressed him with the idea that we had aught to do with its invention, such result was not intended. We were then wholly ignorant of its origin and history. Dr. A. requested the use of it as a pattern, and sent it to Dr. White. Feeling the necessity of a name, and not knowing what else to call it, we suppose Dr. W. named it as above. Not content with the misnomer, we have traced the history of the instrument, and for the present, have the original in our possession.

This instrument was made, about forty-five years ago, for Dr. Hendrick, of Milford, Ohio, in accordance with his own directions, he not being content to extract teeth with only such facilities as were then afforded to physicians. The instrument was improved from time to time, till it reached its present state. Some of these improvements were suggested by Dr. L. A. Hendrick, a well-known dentist, and son of the inventor, some by Dr. W. M. Hunter, Mr. Sherwood, Jno. T. Toland and perhaps others.

In its present state, the instrument is well nigh perfect. It has no sharp angles or corners to lacerate the soft parts of the mouth. Spongy gums are pushed before it, not cut by it. It is perfectly smooth in all its parts, so that it does not roughen a tooth against which it rests as a fulcrum. Almost any root can be removed by it; and very many of them with less pain than is caused by the forceps in seizing them preparatory to extraction. The lower wisdom teeth, when badly decayed, can be removed with it as readily as with the Physic forcep, and much more readily when the face is swollen, and the mouth is opened with difficulty.

A tooth to use as a fulcrum is necessary; but this need not be the one next adjacent to the root to be extracted. Nor need it stand very firmly; as there is but little strain on it. A slight rotation of the instrument, after pressure has commenced, completes the operation.

Many operators are prejudiced against elevators. In abandoning the turnkey for the forceps, the change for the better was so apparent, it is not strange that the inclination is to make the forceps do all. But we are very sure that a skillful operator, with HENDRICKS' ELEVATOR, will remove more than half the roots that need extraction, with less pain than will be inflicted by the forceps in grasping them ; and, at the same time, the gums will not be lacerated to so great an extent. This is important, for the extraction of a tooth is one of the most painful operations known to surgery. The man who, from defective instruments, or want of skill, fails to perform it properly, deserves to be, and should be shunned by the public. W.

